# An eHealth Framework for Managing Pediatric Growth Disorders and Growth Hormone Therapy

**DOI:** 10.2196/27446

**Published:** 2021-05-20

**Authors:** Paul Dimitri, Luis Fernandez-Luque, Indraneel Banerjee, Ignacio Bergadá, Luis Eduardo Calliari, Jovanna Dahlgren, Antonio de Arriba, Risto Lapatto, Thomas Reinehr, Senthil Senniappan, Cécile Thomas-Teinturier, Meng-Che Tsai, Azriyanti Anuar Zaini, Merat Bagha, Ekaterina Koledova

**Affiliations:** 1 The Academic Unit of Child Health Sheffield Children's NHS Foundation Trust Sheffield United Kingdom; 2 Adhera Health Inc Palo Alto, CA United States; 3 Royal Manchester Children’s Hospital Manchester University Hospitals Foundation Trust Manchester United Kingdom; 4 Centro de Investigaciones Endocrinológicas “Dr. César Bergadá” (CEDIE) Hospital de Niños Ricardo Gutiérrez Buenos Aires Argentina; 5 Department of Paediatrics Santa Casa de São Paulo School of Medical Sciences São Paulo Brazil; 6 Department of Pediatrics Region Västra Götaland Sahlgrenska University Hospital Gothenburg Sweden; 7 Department of Pediatrics Institute of Clinical Sciences University of Gothenburg Gothenburg Sweden; 8 Paediatric Endocrinology Hospital Universitario Miguel Servet Zaragoza Spain; 9 New Children’s Hospital University of Helsinki and Helsinki University Hospital Helsinki Finland; 10 Vestische Hospital for Children and Adolescents University of Witten/Herdecke Datteln Germany; 11 Department of Paediatric Endocrinology Alder Hey Children's Hospital Liverpool United Kingdom; 12 Department of Pediatric Endocrinology Assistance Publique – Hôpitaux de Paris, Université Paris Saclay Hôpital Bicetre Le Kremlin Bicêtre France; 13 Department of Pediatrics National Cheng Kung University Tainan Taiwan; 14 Department of Pediatrics University Malaya Medical Centre Kuala Lumpur Malaysia; 15 Tiba Medical Inc Beaverton, OR United States; 16 Global Medical Affairs, Cardiometabolic and Endocrinology Merck KGaA Darmstadt Germany

**Keywords:** eHealth tools, pediatric growth disorders, referral and diagnosis, growth hormone therapy, adherence to treatment, workshop discussions, eHealth, pediatrics, growth failure, growth hormone

## Abstract

**Background:**

The use of technology to support health and health care has grown rapidly in the last decade across all ages and medical specialties. Newly developed eHealth tools are being implemented in long-term management of growth failure in children, a low prevalence pediatric endocrine disorder.

**Objective:**

Our objective was to create a framework that can guide future implementation and research on the use of eHealth tools to support patients with growth disorders who require growth hormone therapy.

**Methods:**

A total of 12 pediatric endocrinologists with experience in eHealth, from a wide geographical distribution, participated in a series of online discussions. We summarized the discussions of 3 workshops, conducted during 2020, on the use of eHealth in the management of growth disorders, which were structured to provide insights on existing challenges, opportunities, and solutions for the implementation of eHealth tools across the patient journey, from referral to the end of pediatric therapy.

**Results:**

A total of 815 responses were collected from 2 questionnaire-based activities covering referral and diagnosis of growth disorders, and subsequent growth hormone therapy stages of the patient pathway, relating to physicians, nurses, and patients, parents, or caregivers. We mapped the feedback from those discussions into a framework that we developed as a guide to integration of eHealth tools across the patient journey. Responses focused on improved clinical management, such as growth monitoring and automation of referral for early detection of growth disorders, which could trigger rapid evaluation and diagnosis. Patient support included the use of eHealth for enhanced patient and caregiver communication, better access to educational opportunities, and enhanced medical and psychological support during growth hormone therapy management. Given the potential availability of patient data from connected devices, artificial intelligence can be used to predict adherence and personalize patient support. Providing evidence to demonstrate the value and utility of eHealth tools will ensure that these tools are widely accepted, trusted, and used in clinical practice, but implementation issues (eg, adaptation to specific clinical settings) must be addressed.

**Conclusions:**

The use of eHealth in growth hormone therapy has major potential to improve the management of growth disorders along the patient journey. Combining objective clinical information and patient adherence data is vital in supporting decision-making and the development of new eHealth tools. Involvement of clinicians and patients in the process of integrating such technologies into clinical practice is essential for implementation and developing evidence that eHealth tools can provide value across the patient pathway.

## Introduction

### eHealth Implementation

The implementation of eHealth solutions in clinical practice is growing rapidly [1]. Digitalization of the health sector is bringing many opportunities, such as the collection of patient-generated data [2] and the provision of health services over distance, also known as telehealth [3]. These technological advances are supporting a paradigm shift towards more integrated health care [4]. However, implementing technologies in the highly complex setting of health care delivery faces many challenges, which include both technical and human factors (eg, organizational issues, service integration, usability, legal frameworks, privacy) [5].

### Opportunities of eHealth for Patient Empowerment in Growth Hormone Therapy

Pediatric endocrinology (the management of endocrine disorders in children) has adopted eHealth solutions for decades. For example, diabetes mellitus education uses game-based interventions [6,7], and more recent advanced technologies, such as augmented reality [8] and robotics [9], have been integrated into education and assistive care in diabetes mellitus. These approaches have traditionally looked at chronic conditions that require intensive lifestyle modifications, where lack of adherence to medication has a negative impact, with an increased risk of health complications and hospitalization.

Similarly, one of the areas that may benefit from implementation of novel eHealth tools is the long-term use of growth hormone therapy in the management of childhood growth failure. Growth hormone therapy is indicated for a heterogeneous set of growth disorders; in addition to growth hormone deficiency, approved pediatric indications include Turner syndrome, short stature after being born small for gestational age, Noonan syndrome, Prader-Willi syndrome, *SHOX* (short stature homeobox-containing gene) deficiency, chronic renal failure, and idiopathic short stature, although licensed indications vary by country and growth hormone formulations [10,11].

Importantly, growth hormone affects not only growth of children, but also metabolism, cardiovascular health, bone strength, and quality of life in the short and long term [12-14]. These effects continue into adult life, and a proportion of patients treated as children continue to require growth hormone therapy as adults. During the transition from pediatric to adult care, eHealth tools have been shown to be important in supporting patients to achieve positive outcomes and enhance ongoing engagement with health care [15,16].

The case of growth hormone therapy is somewhat different from that of diabetes because patients do not require intensive or large-scale lifestyle modifications. Because growth hormone therapy is administered by daily injection, the most important self-management behavior is adherence to medication, which affects long-term outcome. Growth hormone therapy for children with short stature must be administered consistently and with a high level of adherence over many years for an effective gain in height and optimal adult height [17]. Therapy is required from early childhood to adolescence; therefore, support and involvement of the patient’s family and caregivers are crucial to achieving the best outcomes. Thus, eHealth tools to support growth hormone therapy must be developed with a holistic approach that involves the family and must also take into consideration physiological, psychological, and developmental changes as children grow, with a view to using these tools over many years [18,19]. Additionally, some of the conditions that are supported with growth hormone therapy are classified as rare diseases (incidence <1 in 2500 [20]), posing extra challenges. Physicians’ knowledge may be limited, which can often lead to delayed referral and diagnosis, and the small number of patients creates difficulties in collection of data to generate large and meaningful data sets unless performed on an international scale. The collection of adherence data is of crucial importance [21] to optimizing adherence and improving adult height [22,23].

**Figure 1:**
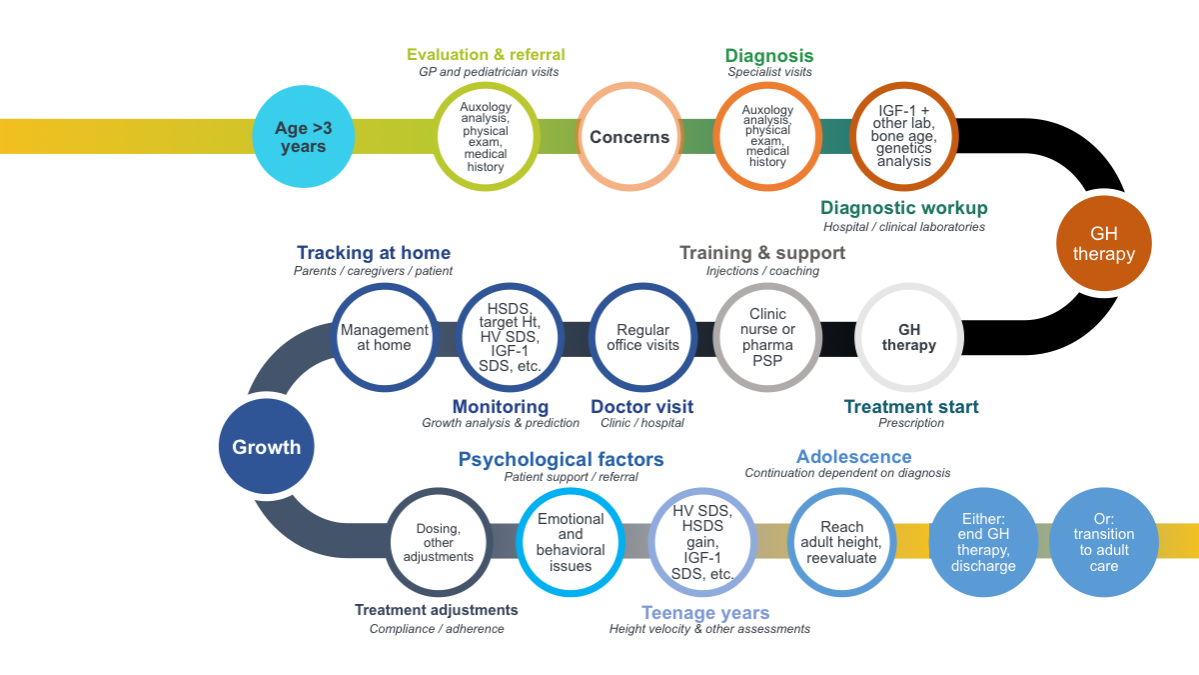
Patient journey for children with growth failure and those who receive growth hormone therapy. GH: growth hormone; GP: general practitioner; HSDS: height standard deviation score; Ht: height; HV: height velocity; IGF-1: insulin-like growth factor-1; PSP: pediatric specialist physician; SDS: standard deviation score.

An optimal adult height is attained through the early recognition and understanding of the different causes of growth failure, thus leading to early referral, diagnosis, and growth hormone therapy initiation. As mapped out in Figure 1, outcomes can be improved at all stages of the patient journey through comprehensive monitoring of growth, education, and clinical support to achieve a high level of therapy adherence. We surmised that eHealth tools could play a significant role in the management of growth disorders and growth hormone therapy through additional and comprehensive support and monitoring.

### Objective

We aimed to develop a framework for using eHealth tools in the identification, investigation and diagnosis of growth failure and the management of growth hormone therapy in pediatric patients. Development of this framework was carried out as a multistep process, guided by the principles of participatory research involving key informants across a series of workshops and virtual activities. Similar approaches have been widely applied in medical informatics to identify implementation challenges of digital health tools [24], including the application of the Delphi method [25] in other endocrine disease areas [26] and eHealth for pediatrics [27].

## Methods

**Figure 2:**
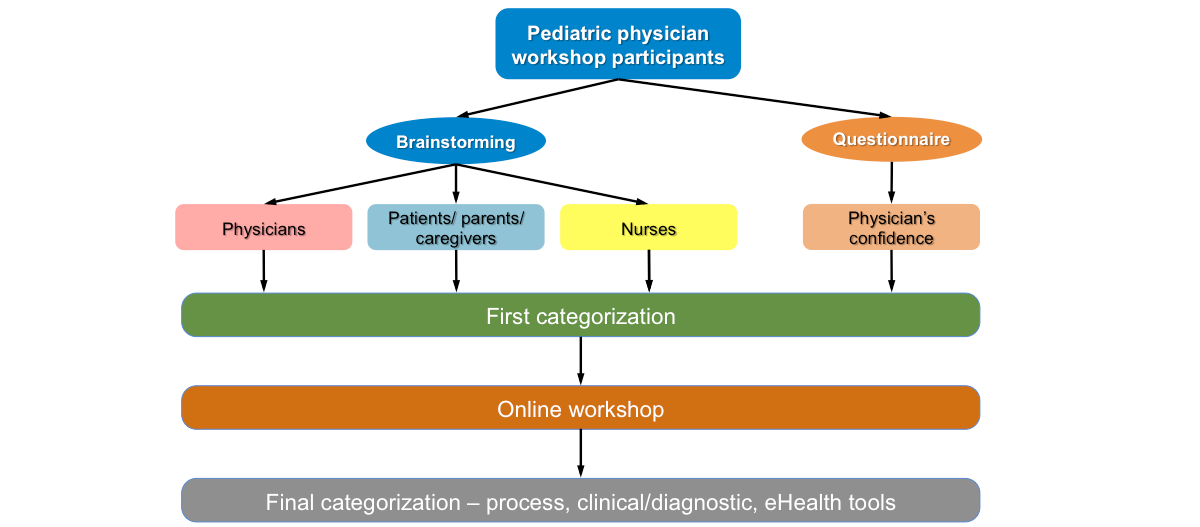
Workshop participation methodological steps. Each step was carried out twice, first in relation to referral and diagnosis stages and second in relation to all stages from growth hormone therapy initiation to completion.

Initially, we conducted 3 online webinars with presentations and interactive discussions around key concepts and issues of eHealth, including data science and use in pediatric patients with growth failure and growth hormone therapy. Based on active involvement in this initial step, a group of 12 participants were subsequently invited to more detailed discussions. This report describes the approach that was adopted for these online detailed discussion workshops and is outlined (Figure 2) and explained in more detail in the following sections.

The 12 participants were pediatric endocrinologists who have experience in the treatment of growth disorders. Convenience sampling was used to select participants on the basis of previous participation, interest or experience in eHealth activities, and patients’ use of connected injection devices for growth hormone therapy. Sampling also considered participants on the basis of providing worldwide geographical representation, with participants from Argentina, Brazil, Finland, France, Germany, Malaysia, Sweden, Spain, Taiwan, and the United Kingdom, encompassing a wide base of health care and socioeconomic conditions. The participants completed preparation activities that included reading relevant publications [28-30], brainstorming activities (see Multimedia Appendix 1), and completing an adapted version of the eHealth literacy scale (eHEALS) survey [31] related to digital health literacy (see Multimedia Appendix 2).

The brainstorming activities solicited opinions of the participants on opportunities, challenges, and established or potential eHealth tools relating to 3 stakeholder groups: (1) pediatric endocrinologists, (2) patients and their parents/caregivers, and (3) nurses. Simultaneously, the pediatric endocrinologists completed a survey about their perception of eHealth tools in aiding clinical decisions and their confidence in use of such tools, with results quantified as proportions of respondents agreeing or disagreeing with the statements on a 5-point scale. The brainstorming activities and surveys were designed using the SurveyMonkey tool (SVMK Inc).

For the first online workshop, the physicians completed the brainstorming exercise and eHealth literacy questionnaire with respect to the first part of the patient journey (Figure 1), through referral and diagnosis. For the second online meeting, the exercise and questionnaire were completed with respect to the subsequent stages of the patient journey from start of growth hormone therapy, through monitoring, tracking, and assessment of outcomes, to either growth hormone discontinuation or into the transition phase from pediatric to adult care for patients still requiring growth hormone therapy.

In each case, the brainstorming responses were initially categorized according to common themes, which were then discussed in the online workshops under the moderation of PD who was the key facilitator and with additional availability of textual responses. The brainstorming responses for each of the 3 categories of opportunities, challenges, and eHealth tools were finally categorized as relating to process, clinical pathway (including diagnostics for the first meeting), or education. These 3 main categories reflected important aspects for the implementation of eHealth solutions in clinical practice and formed the basis for the framework for best practices: (1) process—responses that related to everyday tasks that aided the distance and time logistics required by the specified people for delivery of health care; (2) clinical—responses relating to the medical condition, including those relating to establishing diagnosis; and (3) education—responses relating to digital technologies that can enhance learning and understanding of the condition, and therapy for either health care specialists or the patients and families.

## Results

### Survey Responses

**Figure 3:**
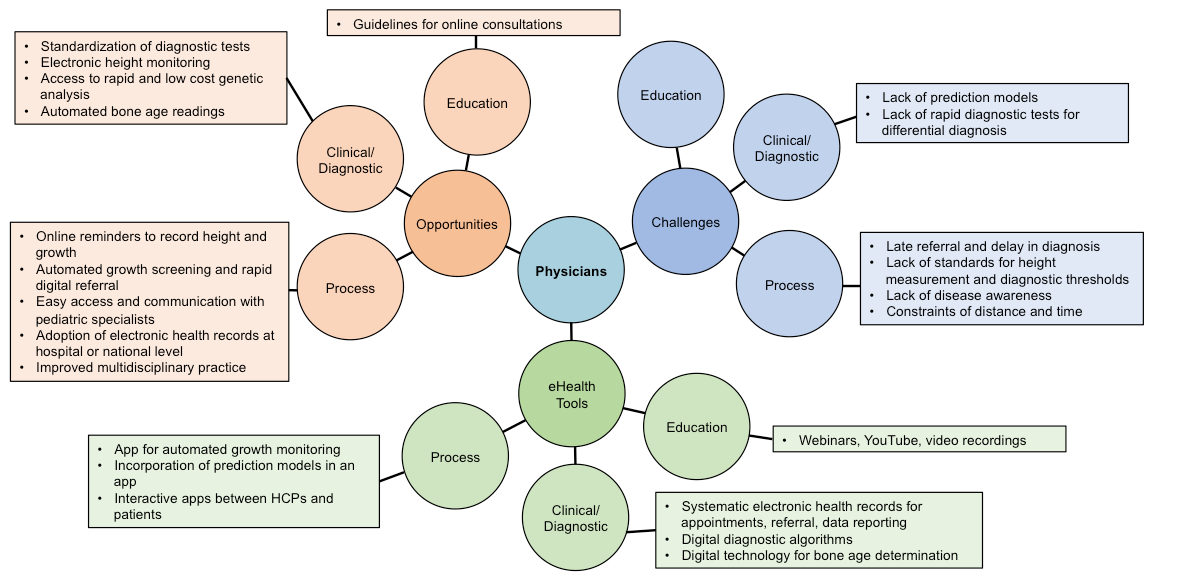
Physician viewpoints regarding referral and diagnosis stages of pediatric patients with growth failure. Where no comments box is shown, clinicians did not provide any opinions relating to that category. HCP: health care provider.

**Figure 4:**
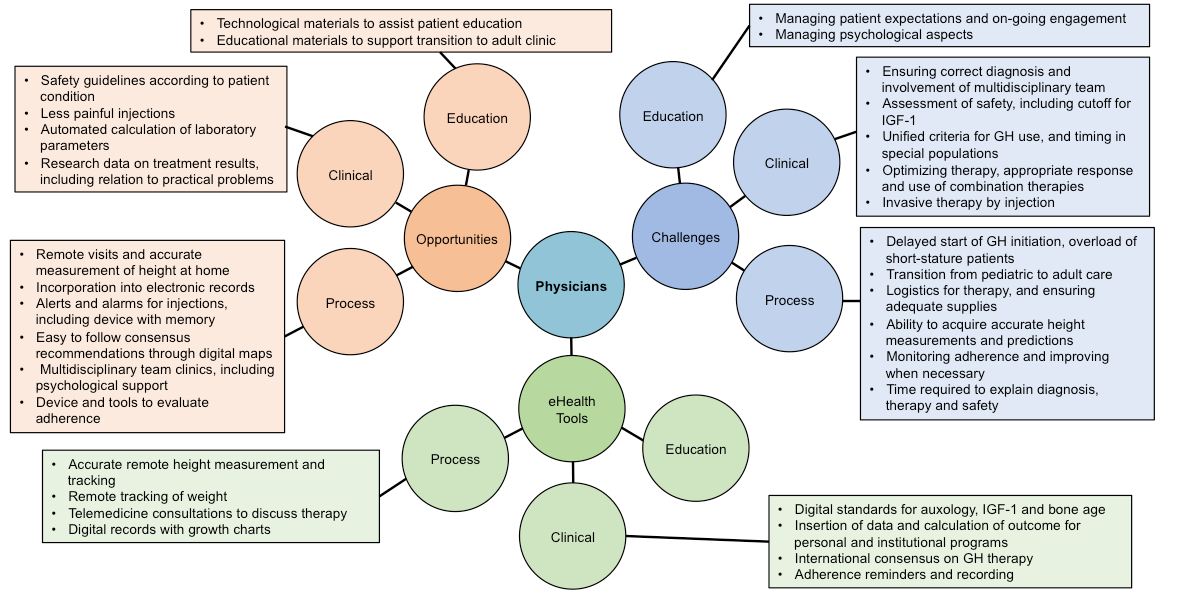
Physician viewpoints regarding growth hormone therapy initiation, monitoring, and transition. Where no comments box is shown, clinicians did not provide any opinions relating to that category. GH: growth hormone; IGF-1: insulin-like growth factor-1.

A total of 815 responses were received from the 2 brainstorming exercises, comprising 419 in relation to referral and diagnosis stages and 396 relating to growth hormone therapy stages. In each case, the responses were grouped and categorized; Figure 3 shows the grouped responses relating to physicians for the referral and diagnosis stages, while Figure 4 shows the growth hormone therapy stages (responses relating to patients/parents/caregivers can be found in Multimedia Appendix 3 for referral/diagnosis and Multimedia Appendix 4 for growth hormone therapy, and responses relating to nurses in Multimedia Appendix 5 for referral/diagnosis and Multimedia Appendix 6 for growth hormone therapy). In the following subsections we describe the responses provided, assigned to each group in turn.

### Brainstorming Responses Relating to Physicians

#### Growth Monitoring and Screening

For the referral and diagnosis stages, the perceived opportunities for physicians largely concerned process, particularly regarding digital reminders to record growth. It was noted that this could be done by primary care personnel, such as in Finland where children’s height is routinely monitored and data immediately entered and integrated into electronic health records. Machine learning or artificial intelligence algorithms could be used to identify faltering growth and be followed by notification of health care professionals of this issue. Although such a system may overestimate referrals, it could support earlier diagnosis of conditions such as Turner syndrome and *SHOX* deficiency; it was noted that physicians may lack knowledge of these types of conditions, and pediatricians may not readily associate signs or symptoms with a diagnosis, resulting in late referral and therapy delay. Algorithms that have been developed to follow through symptomatic characteristics to identify the cause of growth failure could be incorporated into eHealth tools to aid physicians through the early stages of the referral and diagnostic process. Streamlining the screening, referral, and diagnostic process may expedite subsequent genetic testing, particularly for rare conditions associated with growth failure, thus identifying patients eligible for growth hormone therapy at an earlier stage.

#### Automated Height Measurements

At each stage of the patient journey, during referral, diagnosis, and monitoring of response to growth hormone therapy, the ability to accurately measure height electronically was cited as a significant area of development that could overcome the labor-intensive challenge of manually measuring height and inputting this into the patient record. There is potential for height and height velocity to be determined using electronic devices, such as mobile phones or tablet computers, which could be used by health professionals and parents or caregivers. Development of digital techniques for height measurement outside of the clinic would be very helpful in countries with remote rural communities where primary care is lacking, and data could be incorporated directly by linking to electronic health records. A tool that could be used by patients or caregivers to measure height electronically and be linked to artificial intelligence-based technology could increase the frequency of height measurements and provide a signal to health care providers about faltering growth to support earlier intervention.

#### Growth Hormone Response Prediction Models

The response to growth hormone therapy can be determined using validated prediction models that estimate potential adult height by incorporating parameters related to growth. These parameters include bone age, which is a determination of skeletal physiological maturity relative to chronological age through use of hand radiographs that can be evaluated with an automated digital method. There are multiple prediction models and a current lack of consensus regarding which of these is the gold standard. Clinicians wished to see a more unified international approach to growth prediction, which could subsequently lead to the incorporation of a model into an eHealth platform to aid clinicians in determining adult height.

Incorporation of prediction models into apps was considered a challenge, particularly given the lack of consensus on which model to use. It was, however, considered to be an important tool to allow identification of those patients who would most benefit from growth hormone therapy. Moreover, by using a digital device to measure height and then linking this to an electronic patient record, adult height prediction could be provided in a more seamless way through the integration of a prediction model into the process.

#### Communication, Logistics, and Engagement

##### Remote Delivery of Care and Communication

Communication between primary care and pediatric endocrine specialists, and others in multidisciplinary teams, was identified as an opportunity for eHealth tools to improve service provision. Multidisciplinary teams require good videoconferencing facilities; current platforms require improvements, such as increased bandwidth, to facilitate greater use of telemedicine. Guidelines for physicians that augment their ability to carry out online consultations are required, and education facilities for this could be provided through online workshops and video recordings.

One of the biggest challenges mentioned was constraints of time and distance when patients have to attend a hospital or clinic in order for assessments to be made. This may involve considerable travel over long distances (particularly for those in rural settings) and a large amount of time for both patients and health care personnel to ensure that appointments can be initiated and kept. Thus, the refinement of telecommunication systems will help to overcome this barrier.

Participants proposed that electronic health records could be used for automating appointments, referrals, and data reporting. As consultations improve efficiency, more information could be made available for each patient, giving interoperability greater importance in order that data entry does not require additional time. Overall, eHealth tools need to be efficient to use if they are to gain acceptance.

##### Patient Engagement

Digital technologies that can seamlessly record adherence with the therapy regimen allow of evaluation and timely intervention if adherence decreases over time. Adding adherence data to growth prediction models was considered a potential aid to the assessment of patients that would benefit from therapy and could be used as an additional variable in prediction of adult height.

Interactive games that motivate and engage the patient may also increase the long-term adherence to growth hormone therapy, and their development requires involvement of families, nurses, and physicians as key stakeholders.

#### Psychological Well-Being

One challenge for physicians is dealing with the psychological impact of growth hormone therapy. As patients move through different stages of their therapy journey, with evolving physical and emotional maturity, variations in therapy adherence are expected. Digital tools to evaluate the psychological status of patients could enable the identification and assessment of anxiety related to the condition and long-term therapy. Participants suggested that therapy-related psychology could be assessed using short online questionnaires, with standardization across different clinics. In addition, as online and digital tools are developed to deliver psychological interventions, these could be used to support patient care, thus reinforcing therapy adherence.

Evaluation of psychological status is particularly important at the end of pediatric care when patients who remain growth hormone–deficient transition to adult care in order to continue growth hormone therapy to optimize benefits of therapy beyond growth. Digital tools can be used to explain the pathway changes and specific needs to children and young adults, and can also provide feedback to refine care pathways.

#### Safety Assessments

Medical devices should also enable and encourage safety, with mechanisms built into the reporting platforms to individualize according to the specific diagnosis, therapy device, and condition of the patient. The reporting mechanism for safety concerns needs to be carefully considered to determine who should receive and respond to reports. Physicians could also be alerted to any problems in order to differentiate between background symptoms of the condition and adverse events potentially related to therapy.

### Brainstorming Exercise Responses From the Patient/Parent/Caregiver Perspective

#### Patient Education

Physician perspectives on opportunities and eHealth tools relating to patients, parents, or caregivers predominantly focused on education, largely concerning patient awareness, access to trusted information, and increased remote training. Videoconferencing tools are already being used for this to some extent, but more online tools involving apps, websites, medical portals, and search resources are needed to provide information that is accurate, trustworthy (ie, endorsed or produced by reputable sources with knowledge in the area), and easily understood. These tools could also be used to increase access to and interaction with health care personnel; however, access may need to be limited to avoid out-of-hours timing, but this could be mitigated by use of technologies such as virtual assistants or “chatbots ” to provide automated answers.

#### Patient-Reported Outcomes

Digital techniques to accurately measure height at home would enable growth to be monitored without the need for clinical review. These methods could also detect growth deceleration, with integration into electronic health records and prompting alerts sent to patients, parents, or caregivers. Therapy-related anxiety and psychological problems could be aided through games to generate interest in diagnosis and therapy, and to reduce needle anxiety. Appropriate tools could be developed to enhance psychological care, although it was noted that language barriers and digital access problems would need to be overcome to avoid digital exclusion and polarization of patients. Behavioral management may be enhanced through educational tools with a holistic approach, incorporating factors such as good nutrition and exercise to improve metabolic and physical outcomes beyond growth.

### Brainstorming Exercise Responses From the Clinic Nurse Perspective

Responses in regard to nurses, similar to those from physicians, were more often related to process, including support for adherence, and on the medical devices used for therapy delivery. These patient-support tasks are frequently the responsibility of specialized nurses. Difficulties were noted in the delivery of roles, including patient testing, education. and ongoing support, which could be made easier by improved digitalization of and access to records, telemedicine technologies, and remote monitoring; eHealth tools could relieve nurses of some duties, yielding more time for personal interaction with the patient.

Responses also indicated challenges in the receipt and delivery of training and education to nurses around the management of growth failure and therapy, with limited opportunities for learning. In some counties, clinics do not have specialist nurses, and responsibilities may be inadequately defined, with no clear career paths. In such situations, online interactive learning resources could support training.

During growth hormone therapy, virtual reality or augmented reality immersive experiences could help with training and distraction during injections and blood sampling. During transition from pediatric to adult care, digital tools can assist nurses in providing psychological support and education for patients.

### eHealth Literacy Survey

The questionnaire results indicated that the majority of the participants felt that eHealth tools were useful, but some were uncertain about evidence for their usefulness. There was also some uncertainty about what eHealth tools are currently available and how to use them. Most felt that digital tools are important and that they had the skills to use them, but many lacked confidence in their use largely due to the perceived lack of evidence. The respondents indicated that more data on the effectiveness of eHealth tools need to be presented for mainstream use in clinical practice, and interoperability would be required for routine clinical use.

### Pediatric Growth Disorders and Growth Hormone Therapy Management Framework

**Figure 5:**
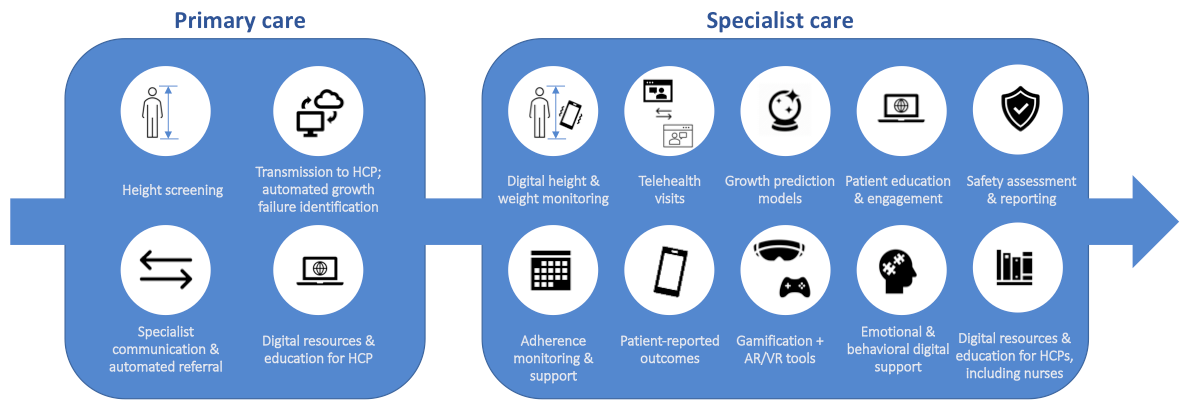
Framework for managing pediatric growth disorders and growth hormone therapy mapped to the patient journey. AR/VR: augmented reality/virtual reality; HCP: healthcare provider.

From the workshop and questionnaire information, we devised a framework for developing eHealth tools for growth disorders and growth hormone therapy, as shown in Figure 5. The framework was devised to support integration of new eHealth tools into health care systems and assist health care professionals, patients, and families throughout the patient journey.

## Discussion

### Principal Findings

We are not aware of any similar studies and, to the best of our knowledge, this is the first time global experts in pediatric endocrinology have come together to consider possible eHealth solutions for the care of children and young people with growth disorders. Our work has led to greater understanding of the acceptability, confidence, and knowledge of the use of eHealth tools in growth disorders, and has opened up digital health care options to improve current clinical management.

Several key themes arose, which were aligned across the themed stakeholder groups considered: physicians, patients/parents/caregivers, and nurses. It was agreed that communication through established and novel technologies are likely to provide significant opportunities to improve interactions between clinicians across the health sector and to facilitate diagnosis, referral, and patient management. Importantly, telecommunication has the potential to improve management of patients who may be in isolated communities or rural areas remote to the central health care provider and to enable parents and caregivers to rapidly communicate with health care professionals when problems arise [32]. Connected digital systems will reduce travel time for families and reduce time of missed education, in turn supporting cost and efficiency savings for families and health services [33]. Integration of these systems into electronic patient records would optimize this process. Additionally, physicians suggested that parents and caregivers would value electronic processes to help them manage appointments, that administration tasks should be automated, and that improved interoperability between systems and within and across centers would allow sharing and integration of vital patient information.

Rapid development of technology could also facilitate the earlier identification of growth disorders through electronic screening programs, with the proviso that the input of height data is accurate. Thus, the development of a mobile phone or tablet-based tool that can accurately measure height in the home environment and that could link with a centralized electronic system may accelerate the identification and referral of patients with short stature and poor growth, reducing the prevalence of late diagnosis of rare disorders of growth where other comorbidities may exist [34-36]. However, a reliable electronic system for early identification of growth disorders is complex and will depend on the integration of systems that monitor height at home and in the clinical environment, with a high sensitivity and specificity [37].

Once a diagnosis of growth failure has been established, an accurate and validated digital tool to monitor height will provide a means of assessing response to growth hormone therapy. Response to growth hormone therapy and improved adherence are both predictors of adult height [23,38,39]. Fundamental to this is the use of artificial intelligence to understand and analyze patient growth data, which can be fed into an electronic health record. When patients become established on growth hormone, clinicians would value a unified means of predicting adult height, encompassing the diverse growth prediction models currently available [40-43]; identifying the impact of growth hormone through prediction models on digital platforms that link to or are embedded in patient records can aid optimization of therapy.

Education was identified as a key area to which eHealth tools could add significant value. Physicians, nurses, and families (including the patients) require relevant and trusted information delivered in an engaging, and developmentally and professionally appropriate way to support the rapid acquisition in knowledge [44,45]. Recent advances in medical education for patients and clinicians have focused on using immersive virtual and augmented reality (AR/VR), app-based interactive training, virtual assistants to answer questions in real-time, 3D modeling, and online real-time education [8,46,47]. In particular, virtual assistants or chatbots have been identified as a technology suitable for supporting patient groups with focused information available in real time [48-50]. One of the challenges in using novel technology-based education is ensuring that hard-to-reach and vulnerable groups have appropriate access to and understanding of these tools [51,52].

Participants also suggested that eHealth tools could be developed to support adherence to growth hormone therapy through the identification of therapy-related psychological and medical issues, potentially via access to data on patient-reported outcome measures [53] or ecological momentary assessments [54]. Given the psychological and physiological changes that take place during growth and puberty, these tools will need to be versatile and responsive, supporting patients and their families during the transition from pediatric to adult health care settings [55]; furthermore, to mitigate the risk of future health complications, such tools need to reiterate education and prevent disengagement from health care [15,16,56]. Importantly, eHealth tools were also cited as an option to address some of the psychological issues identified, with digital platforms providing online psychological support; technologies including VR or AR could provide a means of reducing anxiety during painful procedures such as growth hormone injections, similar to the application of VR and AR as distraction tools in other conditions [8,46,56,57].

Although there was clear support for eHealth solutions to address challenges in the diagnosis and management of patients with growth disorders, results from the e-literacy questionnaire identified a need to ensure that the development and implementation of these tools is supported by a well-established evidence base and that integration within a clinical or home-based setting is supported by education and training.

The workshops and questionnaires have provided information that has led us to devise a framework to support the development of eHealth tools for growth disorders, which may also be relevant to other long-term conditions. The framework (Figure 5) has been devised as an eHealth overlay for the patient journey from identification of a problem, to diagnosis, implementation of therapy, and subsequent monitoring and support. The framework maps directly onto the patient pathway and thus will provide the relevant context for those developing novel technologies. It will support appropriate service and health care integration of new eHealth tools for health care professionals, patients, and families, which in turn will provide reassurance that technology development has appropriately addressed unmet needs for growth disorders.

### Limitations

Participants in this study were chosen for their experience in childhood growth disorders and active interest and involvement in use of eHealth tools. However, participation was limited to physicians, albeit with significant experience in this field of medicine; thus, the views relating to patients, parents, caregivers, and nurses may be skewed by personal reflections and opinions, but responses were perhaps more directed and uniform. The relatively small number that provided responses and opinions may not be wholly representative of the pediatric endocrine community although they represented a wide geographical distribution, allowing us to consider the variation in health care systems in different countries. Due to logistic reasons, face-to-face meetings were not ideal, and we decided to conduct the workshops online, which might have restricted some activities that could have been better conducted through a face-to-face format. Although all participants were highly experienced pediatric endocrinologists, their differing backgrounds meant that there was an inherent variation in cultural perspectives, health care system setups, funding arrangements, and governmental priorities; however, this broad range of experiences could be viewed as reflective of real-world scenarios. Despite all participants having a strong interest in the use of digital techniques, eHealth tools may sometimes be seen to be purely theoretical, as they encompass technologies that could potentially be devised but are not yet established. The integration and adoption of our proposed eHealth framework into clinical practice remains to be assessed and rigorously tested in future studies.

### Conclusions

By bringing together established and experienced pediatric endocrinologists from across the world, we have developed a framework for the implementation and integration of eHealth tools to support the identification and ongoing referral of children with growth disorders, and the subsequent management and monitoring of growth hormone therapy. Integration of eHealth tools in the management of patients with growth disorders will support height screening and regular height monitoring; improve communication; facilitate the delivery of and access to training and education for health care providers, patients, parents, and caregivers; improve therapy adherence; enable the earlier identification of problems; and support patients and families during their journey. Digitalization can enhance the collection of data from multiple sources to support research in low prevalence diseases, such as those treated with growth hormone, to build on best practice by providing an additional incentive for research collaboration using eHealth tools. The use of eHealth tools in the patient pathway has the potential to increase efficiency in care delivery while introducing cost benefits. The involvement of both service providers and service users is of crucial importance for the successful design and implementation of eHealth solutions. The credibility of these eHealth tools will be dependent on appropriate clinical evaluation of established and emerging digital tools. Moreover, training health care professionals and patients and their families in the use of eHealth tools while ensuring successful service integration and ongoing support will prevent the misuse or rejection of eHealth tools and unintended negative health consequences.

We conclude that novel eHealth tools have considerable potential for supporting patients and their families, and for health care providers in the detection and management of growth disorders. We envisage that our work may provide a blueprint for other chronic conditions that could benefit from eHealth tools in supporting long-term adherence.

## Appendix

Multimedia Appendix 1 Online brainstorming assignment. Shown for referral and diagnosis stages and subsequently repeated for growth hormone therapy stages.

Multimedia Appendix 2 eHealth survey. Shown for referral and diagnosis stages and subsequently repeated for growth hormone therapy stages.

Multimedia Appendix 3 Viewpoints on patient and parent/caregiver perspectives for referral and diagnosis stages.

Multimedia Appendix 4 Viewpoints on patient and parent/caregiver perspectives for growth hormone therapy initiation, monitoring and transition stages.

Multimedia Appendix 5 Viewpoints on nurse perspectives for referral and diagnosis stages.

Multimedia Appendix 6 Viewpoints on nurse perspectives for growth hormone therapy initiation, monitoring, and transition stages.

## Abbreviations

AR/VR: augmented reality/virtual reality
eHEALS: eHealth literacy scale
NIH: National Health Service
NIHR: National Institute of Health Research
SHOX: short-stature homeobox-containing gene
