# Supplementary material for: An eHealth Framework for Managing Pediatric Growth Disorders and Growth Hormone Therapy
Source: J Med Internet Res. 2021 May 20;23(5):e27446. doi: 10.2196/27446 (PMC8176345; doi:10.2196/27446)
Supplement: Multimedia Appendix 1 [file jmir_v23i5e27446_app1.docx]

Online brainstorming assignment - Merck Workshop A - final version

Challenges, opportunities and eHealth solutions for improved management of growth hormone treatment

This brainstorming exercise is intended to consider the point of view of people involved in supporting a patient’s growth hormone treatment (GH-Tx) journey, and to better understand their respective challenges, opportunities and related technology solutions – specifically during the Evaluation, Referral and Diagnosis stages.

For this exercise, we ask that you consider the healthcare professionals involved – specifically pediatrician and pediatric endocrinologist, the patient along with his/her parents or caregivers and, separately, the nurse specialists. In this context, what challenges do you think that each of these face at the stages of the patient journey? What opportunities are there to address these challenges? And finally, how can technology solutions and eHealth enable better care with respect to these challenges and opportunities during these stages?

Below are some examples of technologies that might spur your thinking, but please think “outside of the box”, go beyond these and feel free to come up with as many ideas as possible.

The examples are followed by 9 questions. Please provide your opinions on each of the questions, with as many responses as you can think of.


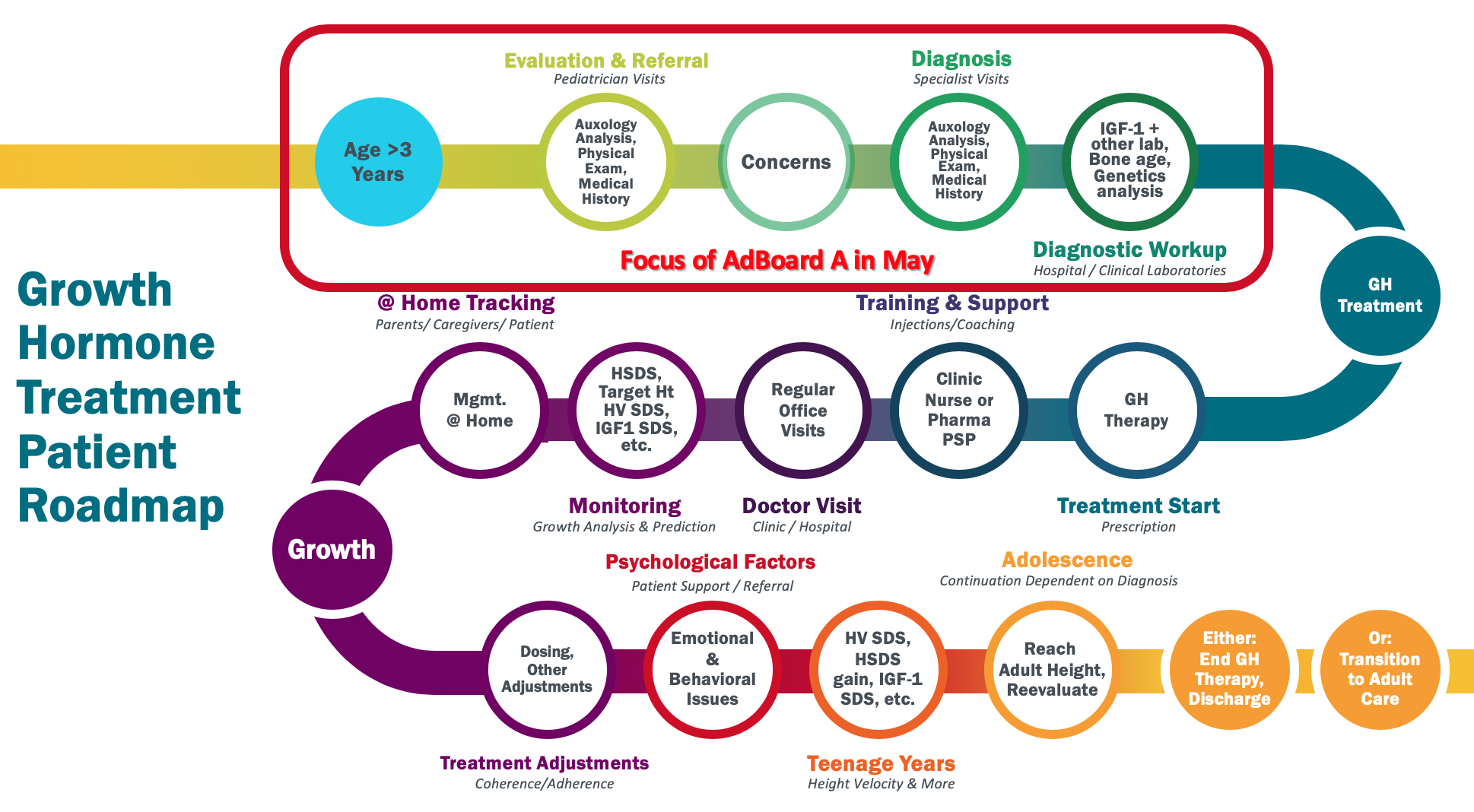


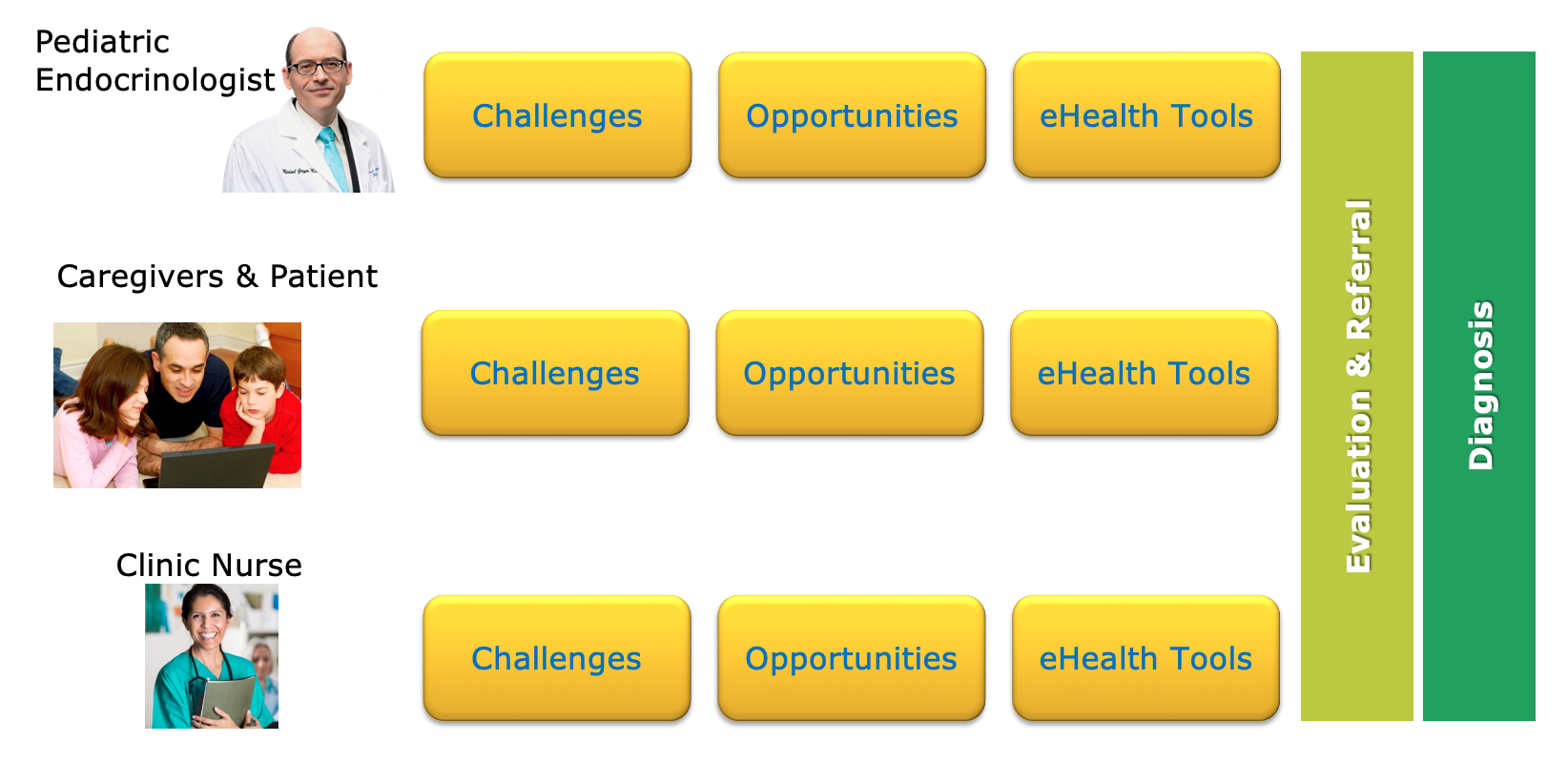


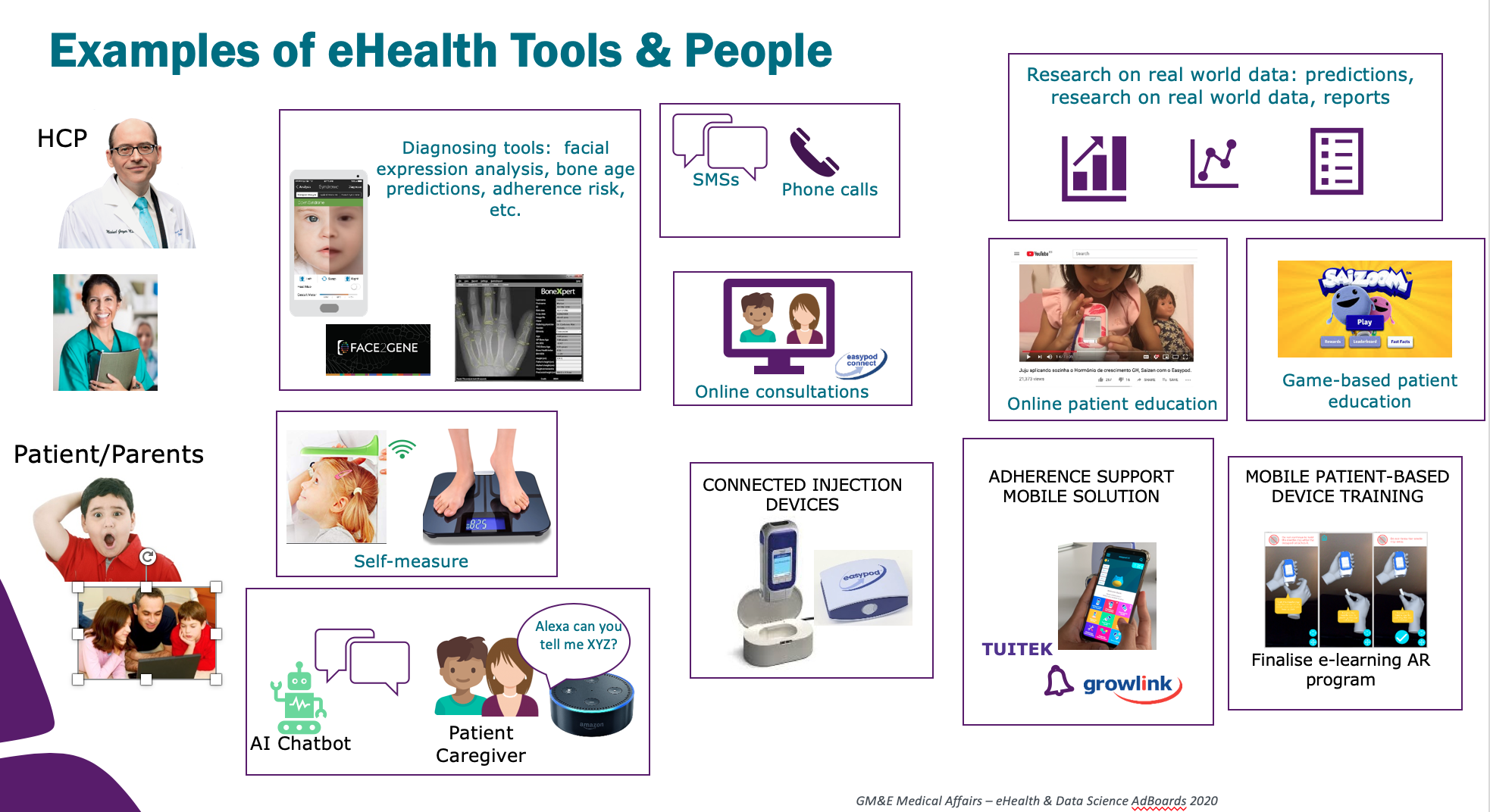


1. What challenges do you and your colleagues face in GH-Tx during the evaluation, referral, and diagnosis stages?

Challenge:

Challenge:

Challenge:

Challenge:

Challenge:

Challenge:

Challenge:

Challenge:

Challenge:

Challenge:

2. What opportunities exist for you and your colleagues in better managing things during the evaluation, referral, and diagnosis stages?

Opportunity:

Opportunity:

Opportunity:

Opportunity:

Opportunity:

3. How and what technology solutions, including eHealth tools, better support you and your colleagues to provide care for GH-Tx during the evaluation, referral, and diagnosis stages?

eHealth tool:

eHealth tool:

eHealth tool:

eHealth tool:

eHealth tool:

4. What about the patient and his/her parents/caregivers?  What challenges do you think they face in GH-Tx during the evaluation, referral, and diagnosis stages?

Challenge:

Challenge:

Challenge:

Challenge:

Challenge:

Challenge:

Challenge:

Challenge:

Challenge:

Challenge:

5. What about the patient and his/her parents/caregivers?  What do you think are opportunities to improve things during the evaluation, referral, and diagnosis stages?

Opportunity:

Opportunity:

Opportunity:

Opportunity:

Opportunity:

6. What technology solutions, including eHealth tools, do you think can better support the patient and his/her parents/caregiver during the evaluation, referral, and diagnosis stages?

eHealth tool:

eHealth tool:

eHealth tool:

eHealth tool:

eHealth tool:

7. What about the clinic nurse?  What do you think about her/his challenges during the evaluation, referral, and diagnosis stages?

Challenge:

Challenge:

Challenge:

Challenge:

Challenge:

Challenge:

Challenge:

Challenge:

Challenge:

Challenge:

8. What opportunities do you think exist for the clinic nurse to improve things during the evaluation, referral, and diagnosis stages?

Opportunity:

Opportunity:

Opportunity:

Opportunity:

Opportunity:

9. With regard to the clinic nurse, how do you think technology solutions, including eHealth tools, can help her/him during the evaluation, referral, and diagnosis stages?

eHealth tool:

eHealth tool:

eHealth tool:

eHealth tool:

eHealth tool:

10. Would you mind providing your email, so we can contact you in case we have some follow up questions about your answers.

Name

Email Address
