# Supplementary material for: An eHealth Framework for Managing Pediatric Growth Disorders and Growth Hormone Therapy
Source: J Med Internet Res. 2021 May 20;23(5):e27446. doi: 10.2196/27446 (PMC8176345; doi:10.2196/27446)
Supplement: Multimedia Appendix 2 [file jmir_v23i5e27446_app2.docx]

eHealth Survey - Merck Workshop A (Evaluation, Referral & Diagnosis Stages)

Knowledge and skills of eHealth Tools across the patient journey

This survey is designed to better understand your perceptions about the use of eHealth tools to paediatric patients with growth hormone disorders undergoing growth hormone treatment.

eHealth tools include mobile solutions, clinical decision support systems (e.g. age bone calculators), and any other electronic and computing device that can be used in the clinical practice for supporting patients. Further, eHealth tools can be used for tracking adherence to the treatment plan, engaging and informing patients and their caregivers, and providing better insights to the healthcare team to further support patients through their GH treatment journey.

In this first round of the survey we focus on two stages of the Growth Hormone Treatment Patient Roadmap (see image below):

1. Evaluation & Referral (e.g. auxology analysis, physical exam, medical history conducted by the pediatrician)
2. Diagnosis (e.g. auxology analysis, physical exam, medical history, plus IGF-1, other laboratory evaluation (e.g. GH stimulation test for GHD, biochemistry to exclude other causes of short stature), assessment of bone age, and genetics analysis (if required), conducted by the pediatric endocrinologist at the clinic or hospital)

Remember, there are no wrong or right answers!

Growth Hormone Treatment Patient Roadmap


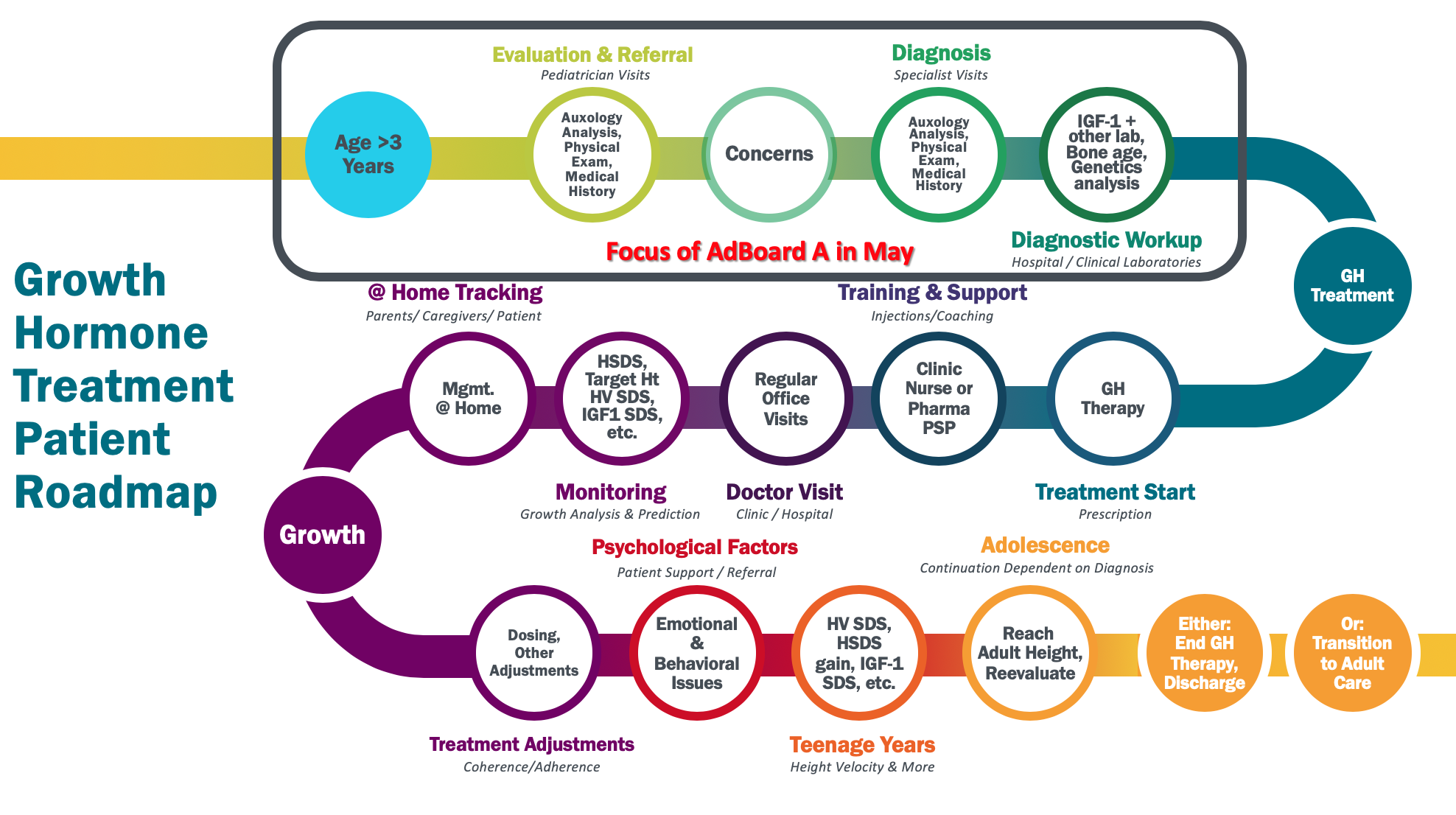


1. How useful do you feel that eHealth tools are in helping you in making decisions?

|  | Not useful at all | Not useful | Unsure | Useful | Very Useful |
| --- | --- | --- | --- | --- | --- |
| at Referral/Evaluation |  |  |  |  |  |
| at Diagnosis |  |  |  |  |  |

2. How important is it for you to be able to use eHealth tools?

|  | Not important at all | Not important | Unsure | Important | Very Important |
| --- | --- | --- | --- | --- | --- |
| at Referral/Evaluation |  |  |  |  |  |
| at Diagnosis |  |  |  |  |  |

3. I have the skills I need to evaluate the eHealth tools to support my clinical decision making

|  | Strongly disagree | Disagree | Unsure | Agree | Strongly agree |
| --- | --- | --- | --- | --- | --- |
| at Referral/Evaluation |  |  |  |  |  |
| at Diagnosis |  |  |  |  |  |

4. I know how to use the eHealth tools to support my clinical decision making

|  | Strongly disagree | Disagree | Unsure | Agree | Strongly agree |
| --- | --- | --- | --- | --- | --- |
| at Referral/Evaluation |  |  |  |  |  |
| at Diagnosis |  |  |  |  |  |

5. I feel confident in using eHealth tools to support my clinical decision making

|  | Strongly disagree | Disagree | Unsure | Agree | Strongly agree |
| --- | --- | --- | --- | --- | --- |
| at Referral/Evaluation |  |  |  |  |  |
| at Diagnosis |  |  |  |  |  |
