# Supplementary material for: An eHealth Framework for Managing Pediatric Growth Disorders and Growth Hormone Therapy
Source: J Med Internet Res. 2021 May 20;23(5):e27446. doi: 10.2196/27446 (PMC8176345; doi:10.2196/27446)
Supplement: Multimedia Appendix 3 [file jmir_v23i5e27446_app3.pptx]

## Slide 1
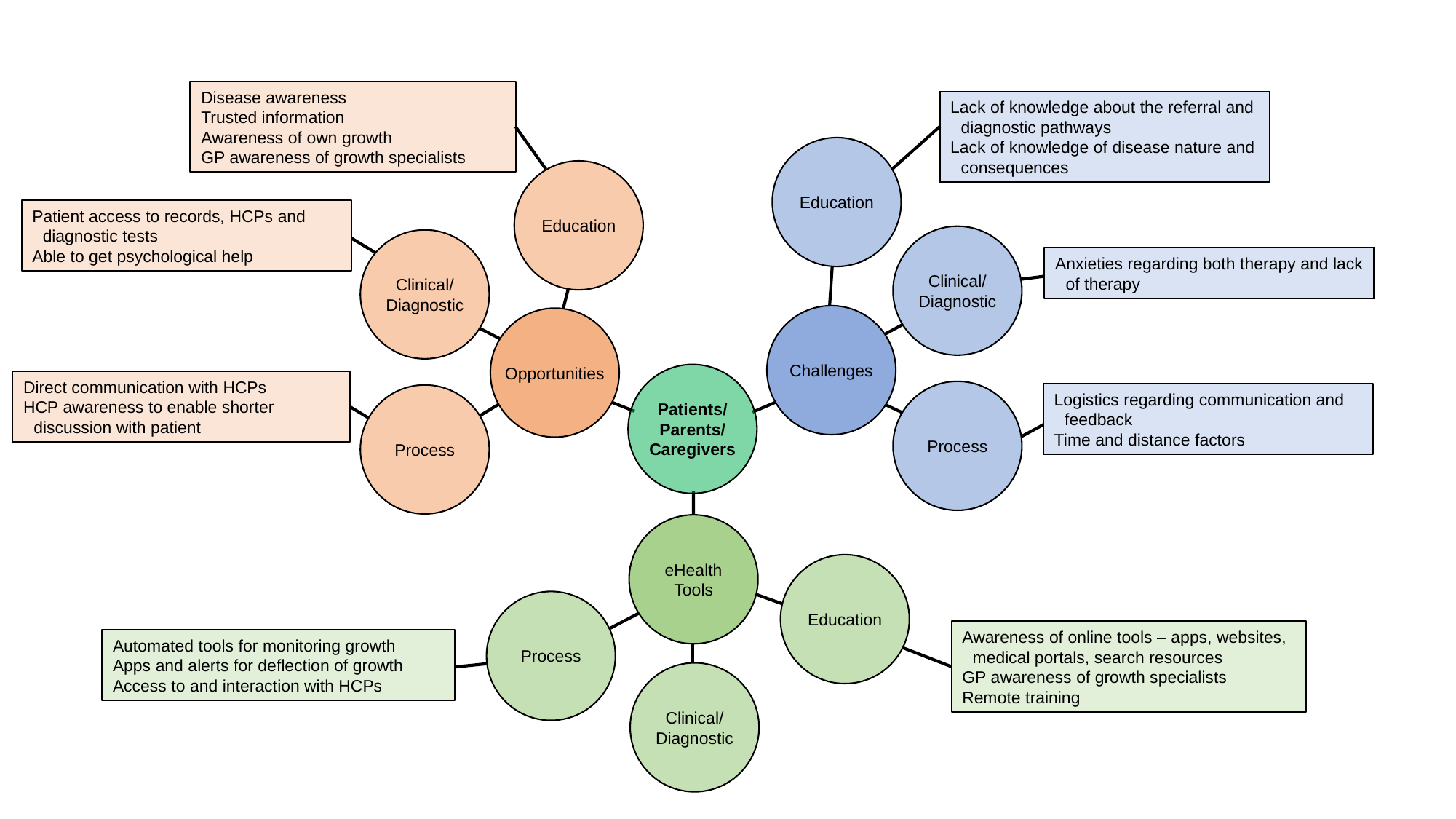

Disease awareness
Trusted information
Awareness of own growth
GP awareness of growth specialists
Lack of knowledge about the referral and diagnostic pathways
Lack of knowledge of disease nature and consequences
Education
Education
Patient access to records, HCPs and diagnostic tests
Able to get psychological help
Clinical/ Diagnostic
Clinical/ Diagnostic
Anxieties regarding both therapy and lack of therapy
Challenges
Opportunities
Patients/ Parents/ Caregivers
Direct communication with HCPs
HCP awareness to enable shorter discussion with patient
Process
Logistics regarding communication and feedback
Time and distance factors
Process
eHealth Tools
Education
Process
Awareness of online tools – apps, websites, medical portals, search resources
GP awareness of growth specialists
Remote training
Automated tools for monitoring growth
Apps and alerts for deflection of growth
Access to and interaction with HCPs
Clinical/ Diagnostic
