# Supplementary material for: An eHealth Framework for Managing Pediatric Growth Disorders and Growth Hormone Therapy
Source: J Med Internet Res. 2021 May 20;23(5):e27446. doi: 10.2196/27446 (PMC8176345; doi:10.2196/27446)
Supplement: Multimedia Appendix 4 [file jmir_v23i5e27446_app4.pptx]

## Slide 1
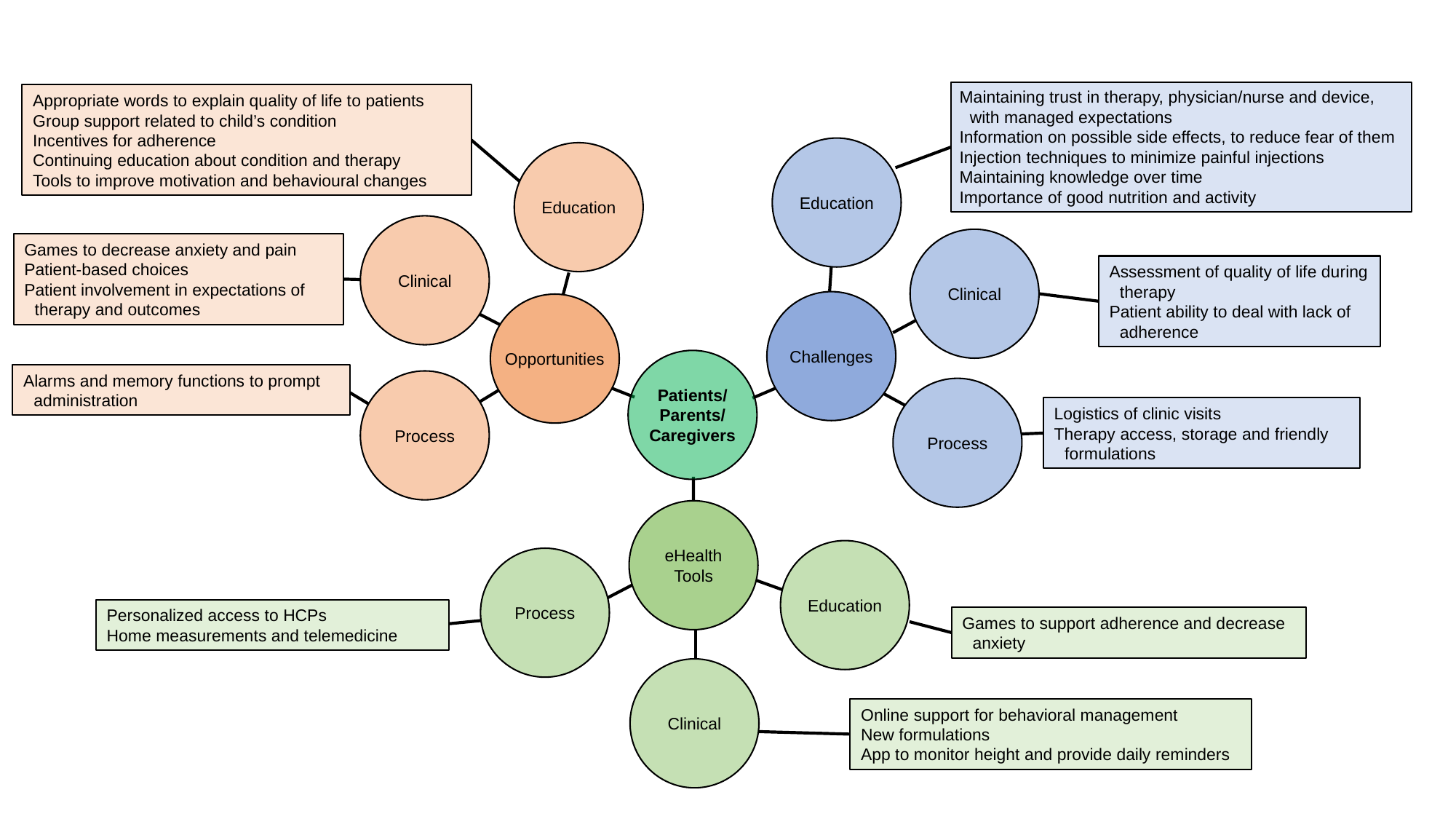

Maintaining trust in therapy, physician/nurse and device, with managed expectations
Information on possible side effects, to reduce fear of them
Injection techniques to minimize painful injections
Maintaining knowledge over time
Importance of good nutrition and activity
Appropriate words to explain quality of life to patients
Group support related to child’s condition
Incentives for adherence
Continuing education about condition and therapy
Tools to improve motivation and behavioural changes
Education
Education
Clinical
Clinical
Games to decrease anxiety and pain
Patient-based choices
Patient involvement in expectations of therapy and outcomes
Assessment of quality of life during therapy
Patient ability to deal with lack of adherence
Challenges
Opportunities
Patients/ Parents/ Caregivers
Alarms and memory functions to prompt administration
Process
Process
Logistics of clinic visits
Therapy access, storage and friendly formulations
eHealth Tools
Education
Process
Personalized access to HCPs
Home measurements and telemedicine
Games to support adherence and decrease anxiety
Clinical
Online support for behavioral management
New formulations
App to monitor height and provide daily reminders
