# Supplementary material for: An eHealth Framework for Managing Pediatric Growth Disorders and Growth Hormone Therapy
Source: J Med Internet Res. 2021 May 20;23(5):e27446. doi: 10.2196/27446 (PMC8176345; doi:10.2196/27446)
Supplement: Multimedia Appendix 5 [file jmir_v23i5e27446_app5.pptx]

## Slide 1
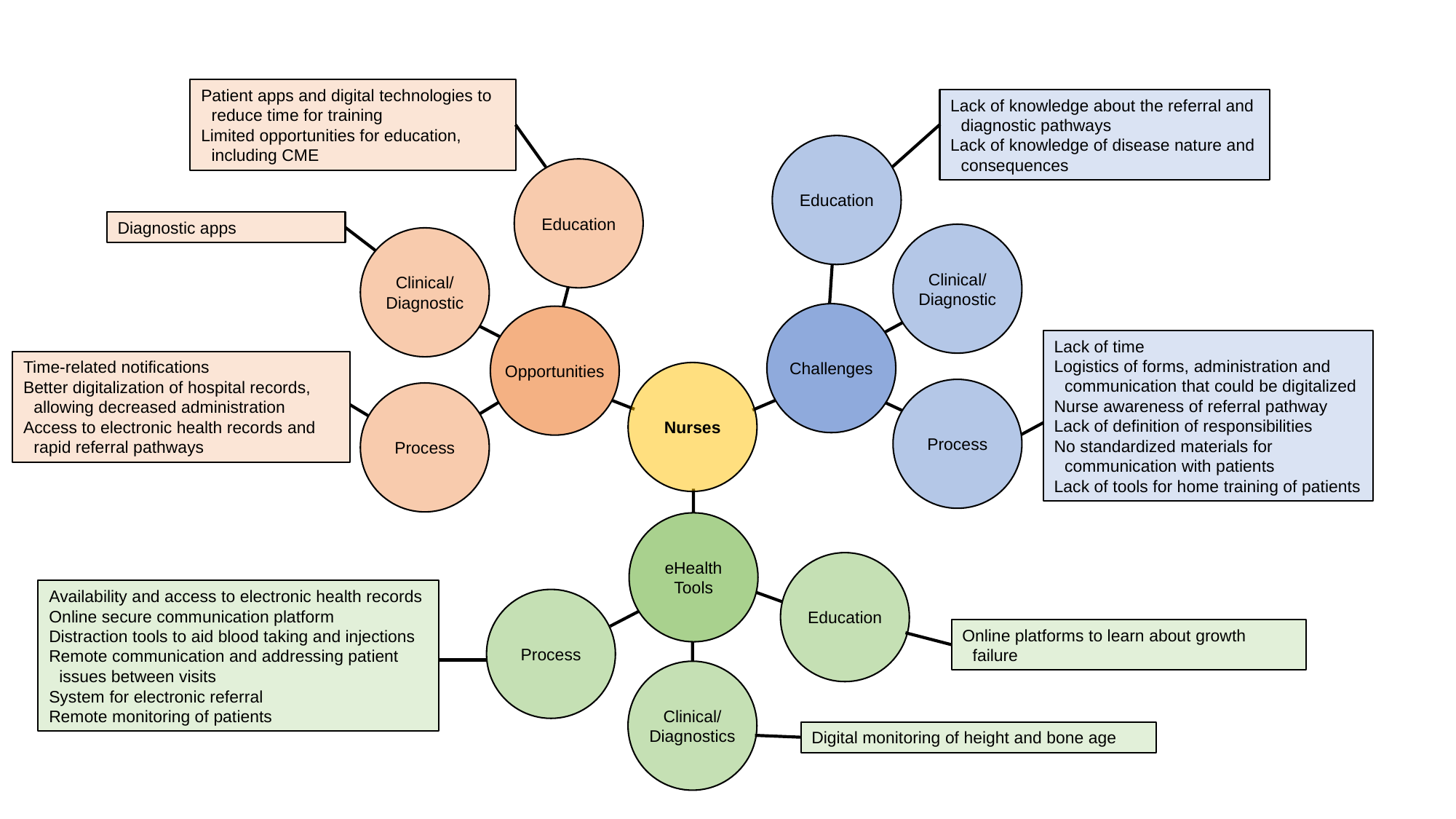

Patient apps and digital technologies to reduce time for training
Limited opportunities for education, including CME
Lack of knowledge about the referral and diagnostic pathways
Lack of knowledge of disease nature and consequences
Education
Education
Diagnostic apps
Clinical/ Diagnostic
Clinical/ Diagnostic
Challenges
Opportunities
Lack of time
Logistics of forms, administration and communication that could be digitalized
Nurse awareness of referral pathway
Lack of definition of responsibilities
No standardized materials for communication with patients
Lack of tools for home training of patients
Time-related notifications
Better digitalization of hospital records, allowing decreased administration
Access to electronic health records and rapid referral pathways
Nurses
Process
Process
eHealth Tools
Education
Availability and access to electronic health records
Online secure communication platform
Distraction tools to aid blood taking and injections
Remote communication and addressing patient issues between visits
System for electronic referral
Remote monitoring of patients
Process
Online platforms to learn about growth failure
Clinical/ Diagnostics
Digital monitoring of height and bone age
