# Supplementary material for: An eHealth Framework for Managing Pediatric Growth Disorders and Growth Hormone Therapy
Source: J Med Internet Res. 2021 May 20;23(5):e27446. doi: 10.2196/27446 (PMC8176345; doi:10.2196/27446)
Supplement: Multimedia Appendix 6 [file jmir_v23i5e27446_app6.pptx]

## Slide 1
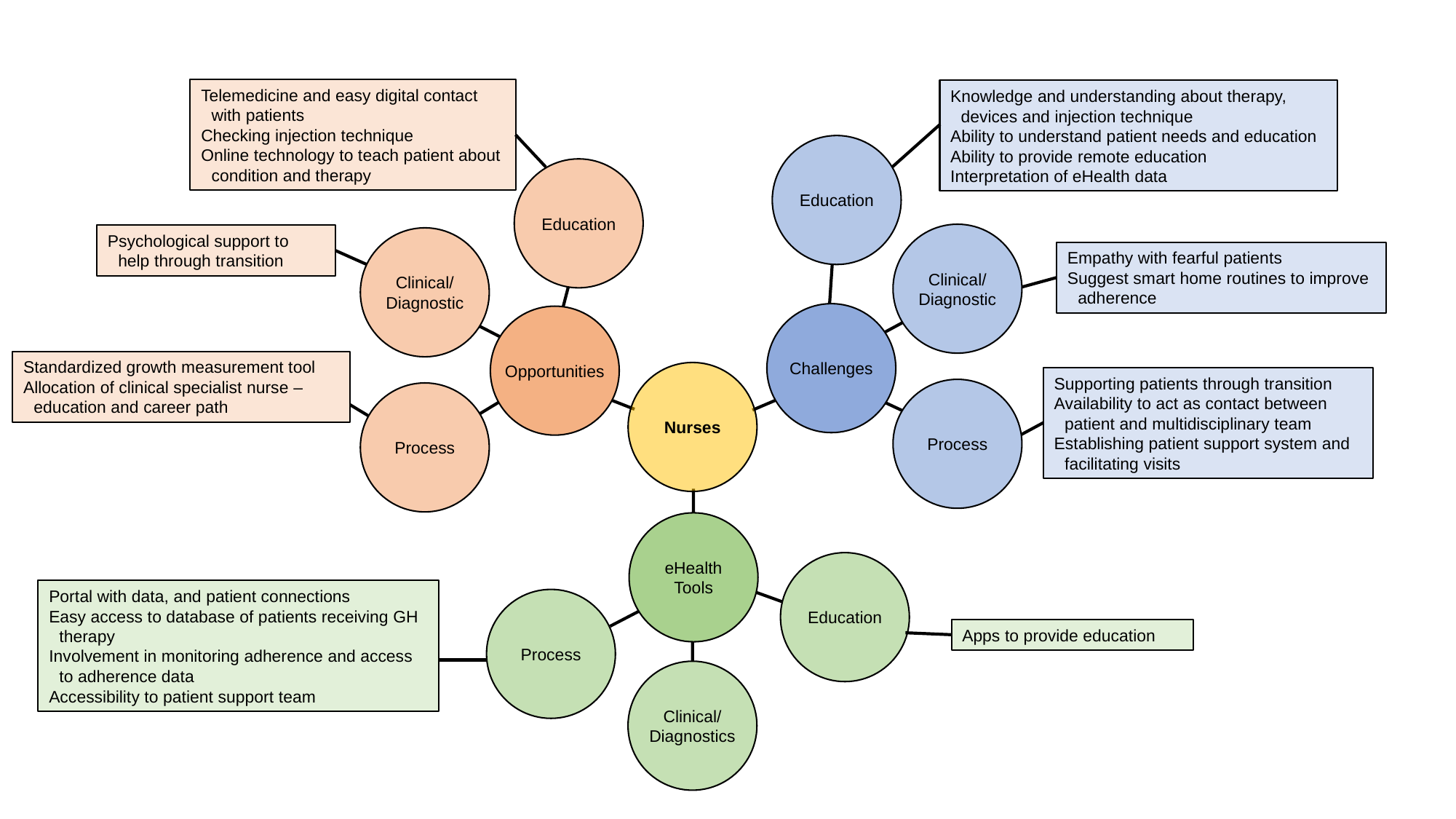

Telemedicine and easy digital contact with patients
Checking injection technique
Online technology to teach patient about condition and therapy
Knowledge and understanding about therapy, devices and injection technique
Ability to understand patient needs and education
Ability to provide remote education
Interpretation of eHealth data
Education
Education
Clinical/ Diagnostic
Psychological support to help through transition
Clinical/ Diagnostic
Empathy with fearful patients
Suggest smart home routines to improve adherence
Challenges
Opportunities
Standardized growth measurement tool
Allocation of clinical specialist nurse – education and career path
Nurses
Supporting patients through transition
Availability to act as contact between patient and multidisciplinary team
Establishing patient support system and facilitating visits
Process
Process
eHealth Tools
Education
Portal with data, and patient connections
Easy access to database of patients receiving GH therapy
Involvement in monitoring adherence and access to adherence data
Accessibility to patient support team
Process
Apps to provide education
Clinical/ Diagnostics
